# Preclinical Efficacy and Proteomic Prediction of Molecular Targets for s-cal14.1b and s-cal14.2b Conotoxins with Antitumor Capacity in Xenografts of Malignant Pleural Mesothelioma

**DOI:** 10.3390/md23010032

**Published:** 2025-01-10

**Authors:** Angélica Luna-Nophal, Fernando Díaz-Castillo, Vanessa Izquierdo-Sánchez, Jesús B. Velázquez-Fernández, Mario Orozco-Morales, Luis Lara-Mejía, Johana Bernáldez-Sarabia, Noemí Sánchez-Campos, Oscar Arrieta, José Díaz-Chávez, Jorge-Ismael Castañeda-Sánchez, Alexei-Fedorovish Licea-Navarro, Saé Muñiz-Hernández

**Affiliations:** 1 Doctorado en Ciencias Biológicas y de la Salud, Universidad Autónoma Metropolitana, Ciudad de Mexico 04960, Mexico; angitaluna87@gmail.com; 2 Laboratorio de Oncología Experimental, Subdirección de Investigación Básica, Instituto Nacional de Cancerología, Ciudad de Mexico 14080, Mexico; orozco81@hotmail.com (M.O.-M.); ogar@unam.mx (O.A.); van.izq.san@gmail.com (V.I.-S.); 3 Departamento de Innovación Biomédica, Centro de Investigación Científica y de Educación Superior de Ensenada (CICESE), Ensenada 22860, Mexico; ferdiaz@cicese.edu.mx (F.D.-C.); jbernald@cicese.edu.mx (J.B.-S.); lsanchez@cicese.edu.mx (N.S.-C.); 4 Control de Calidad, Unidad Ciclotrón & Radiofarmacia, Instituto Nacional de Cancerología, Ciudad de Mexico 14080, Mexico; 5 CONAHCyT-Investigador por Mexico, Ciudad de Mexico 14080, Mexico; jesusbvf@gmail.com; 6 Departamento de Biotecnología y Bioingeniería, Centro de Investigación y de Estudios Avanzados—IPN, Ciudad de Mexico 07360, Mexico; 7 Laboratorio Neuroinmunología, Instituto Nacional de Neurología y Neurocirugía, Ciudad de Mexico 14269, Mexico; 8 Unidad de Oncología Torácica, Instituto Nacional de Cancerología, Ciudad de Mexico 14080, Mexico; dr.luislaramejia@gmail.com; 9 Laboratorio de Oncologia Molecular y Biomarcadores, Subdirección de Investigación Básica, Instituto Nacional de Cancerología, Ciudad de Mexico 14080, Mexico; josediaz030178@hotmail.com; 10 Tecnológico de Monterrey, Escuela de Medicina y Ciencias de la Salud, Ciudad de Mexico 14380, Mexico; 11 Departamento de Sistemas Biológicos, Universidad Autónoma Metropolitana, Unidad Xochimilco, Ciudad de Mexico 04960, Mexico; jcastanedas@correo.xoc.uam.mx

**Keywords:** pharmacological activity, therapeutic effect, mass spectrometry, marine toxins, protein markers

## Abstract

Malignant pleural mesothelioma (MPM) is a rare neoplasm with increasing incidence and mortality rates. Although recent advances have improved the overall prognosis, they have not had an important impact on survival of patients with MPM, such that more effective treatments are needed. Some species of marine snails have been demonstrated to be potential sources of novel anticancer molecules. This study analyzed the anticancer effects in vitro and in vivo of two peptides found in *C. californicus*. The effects of s-cal14.1b and s-cal14.2b on cell proliferation, apoptosis, and cytotoxicity were evaluated in 2D and 3D cultures of MPM-derived cells. Proteomics analysis of 3D cultures treated with conotoxins was performed to examine changes in expression or abundance. And the therapeutic effects of both conotoxins were evaluated in MPM mouse xenografts. s-cal14.1b and s-cal14.2b induced apoptosis and cytotoxicity in 2D and 3D cultures. However, only s-cal14.1b modified spheroid growth. Approximately 600 proteins exhibited important differential expression, which was more heterogeneous in H2452 vs MSTO-211H spheroids. The in silico protein functional analysis showed modifications in the biological pathways associated with carcinogenesis. CAPN1, LIMA1, ANXA6, HUWE1, PARP1 or PARP4 proteins could be potential cell targets for conotoxins and serve as biomarkers in MPM. Finally, we found that both conotoxins reduced the tumor mass in MPM xenografts; s-cal14.1b reached statistical significance. Based on these results, s-cal14.1b and s-cal14.2b conotoxins could be potential therapeutic drugs for MPM neoplasms with no apparent side effects on normal cells.

## 1. Introduction

Malignant pleural mesothelioma (MPM) is a rare neoplasm with a high mortality rate and is predominantly associated with asbestos exposure. Although in the 1980s the use of this mineral was prohibited, it is still used in several industries worldwide [1]. According to data from the World Health Organization (WHO), in 2019 mesothelioma recorded nearly 31,000 new cases and 26,278 deaths, of which 41.3% originated in pleural lung [2]. Generally, deaths by MPM occur in developed countries [2,3]; this is probably related to a better registration process than in less developed nations; in 59 countries with good quality recorded data, more than 15,000 deaths/year are recorded [4]. In 2011, at least 25.9% of deaths related to MPM occurred in the American continent [3], and in Mexico, this neoplasia ranks 34th among all neoplasms despite the under-registration, and it is assumed to account for up to 70% of total cases [5,6].

Since 2004, the FDA approved the pemetrexed/cis- or carboplatin in combination as a first-line regimen therapeutic, with a median of time of overall survival of 12.7–14 moths. Agents such as gemcitabine, vinorelbine, liposomal doxorubicin, and gefitinib between others therapeutic agents have been used for a long time with limited efficacy [7,8,9,10,11,12]. Although the introduction of immunotherapy has changed the first-line treatment landscape of MPM, especially for the sarcomatoid histology, the overall prognosis of most patients remains dismal. In the recently published Checkmate 743 study, the overall survival (OS) with the nivolumab plus ipilimumab arm was 18.1 months, a 4-month advantage compared with platinum plus pemetrexed [13]. On the other hand, molecules of natural origin, such as toxins of plant and animal origin, have become therapeutic alternatives for the treatment of several diseases [14,15]. Molecules from other marine sources, including algae, sponges, coral, and cone snails, are still in the research stage, with promising outcomes [16].

Marine cone snails are a diverse group of animals comprising 500–700 species [17]. Conotoxins are short peptides of 8–50 amino acids that are cysteine-rich and characteristically target-selective for a large number of receptors and ion channel proteins [17,18]. Some conotoxins have well known therapeutic applications, such as ω-conotoxin MVIIA (known as ziconotide), which is currently used to treat chronic pain in patients with AIDS or cancer [19]. *Californiconus californicus* is a snail species endemic to California (USA) and Baja California (Mexico); molecular analysis has placed it in a site phylogenetically distant from other marine snails; so, the toxins of this species represent a unique source of peptides with potential pharmacological activity [19,20]. Most of these conopeptides, have a sequence of 17 amino acids, with several cysteines, which gives them structural stability. Particularly, in previous studies by our group and others, using peptides isolated from marine organisms such as *C. californicus* has shown an inhibitory effect on cancer cells, impacting survival, angiogenesis, apoptosis induction, and cytoskeleton modifications, among other mechanisms; however, research concerning their interaction with cell target proteins is still lacking [21,22,23]. Moreover, in MPM there is no information about the probable antitumor effect of conotoxins.

Proteomics benefits from mass spectrometry techniques for detecting and comparing a large number of differentially expressed proteins [24]. Indeed, bottom-up proteomics is a state-of-art approach for analyzing and understanding how the whole set of proteins in tissues may be affected by other organisms or compounds, by determining relative protein abundances and their variation upon a condition such as cancer or treatment with certain candidate drugs [24]. The 3D models, such as spheroids, show a particular biology compared to 2D or cellular monolayer cultures; for instance, their intake of nutrients by means of a gradient from the outside and their growth linked to cell–cell interactions lead to the formation of a structure that mimics an in vivo tumor more precisely than 2D cultures [25].

Thus, to understand the role of conotoxins on cancer treatment, this work aimed to determine the effect of s-cal14.1b and s-cal14.2b from *Californiconus californicus* upon cell cultures in monolayers (2D), spheroids (3D), and MPM xenograft model, as well as to analyze proteomes from 3D cultures treated with such conopeptides.

## 2. Results

### 2.1. s-cal14.2b Had Larger Effect on Proliferation of Epithelial Mesothelioma-Derived Cell Line

**Figure 1:**
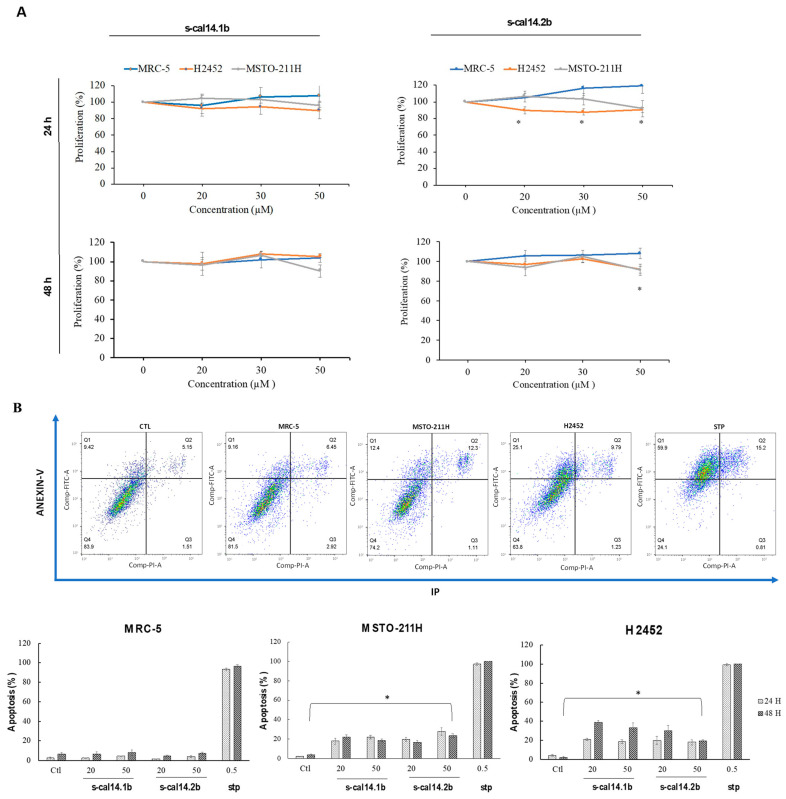
(**A**) Percentages of proliferation obtained at 24 h and 48 h post-exposition with s-cal14.1b and s-cal12.2b toxin in monolayers cultures. * The point where *p* = 0.05, compared the treated vs untreated monolayer. (**B**) At the top are present plots representative of apoptosis acquisition and at the bottom are present apoptosis percentage graphs. MRC-5 cell lines do not have a statistically significant induction of apoptosis. Percentage of apoptosis induced in H2452 and MSTO-211H cell lines reaches a statistical significance. * Statistical significance *p* ≤ 0.05.

Proliferation assays on monolayer cultures were performed to determine whether s-cal14.1b or s-cal14.2b inhibited cell proliferation. The cell line derived from normal lung; MRC-5 did not show proliferative inhibition by any conotoxin at any exposure time (Figure 1A, blue lines). The conotoxin s-cal14.1b did not show an inhibitory effect on any cell line (Figure 1A, s-cal14.1b). In contrast, s-cal14.2b inhibited cellular proliferation in H2452 cells at 24 h at all the assessed concentrations (Figure 1A, orange lines, asterisks). While, in MSTO-211H cells, only the maximum conotoxin concentration showed a significant inhibitory effect (Figure 1A, gray lines). At 48 h post-exposure, both the H2452 and MSTO-211H cells showed proliferation inhibitory at the higher toxin concentration (Figure 1A s-cal14.2b, asterisk).

### 2.2. Apoptosis Was Induced in Cell Line Monolayers After s-cal14.1b and s-cal14.2b Exposition

In monolayers of normal cells, MRC-5, no induction of apoptosis was observed under any experimental condition (Figure 1B, MRC-5). MSTO-211H monolayers showed no differences in the apoptosis induction at both conotoxins and two exposure times (Figure 1B; MSTO-211H), whereas apoptosis was induced by both conotoxins in the H2452 cell monolayers, which was higher at 48 h; however, no differences between 24 and 48 h were reported with the s-cal14.2b conotoxin at 50 µM (Figure 1B; H2452).

### 2.3. s-cal14.1b but Not s-cal14.2b Impaired Growth and Morphology of Multicellular Spheroids

Because s-ca14.1b and s-cal14.2b affected both proliferation and apoptosis in monolayers, we determine whether they had any effect on 3D cultures. Based on the effect observed in proliferation, from here on we used a concentration of 50 µM of s-cal14.2b; in all assays with MSTO-211H cell line, for the s-cal14.1b and H2452 cell line, the concentration used was 20 µM. Also, we used two time periods: the formation period, comprising the first five days of cultivation (1–5 days), and the growth period, the days afterward (6–14 days), to evaluate the conotoxin effects in spheroid cultures (detailed in the “Materials and Methods” section).

**Figure 2:**
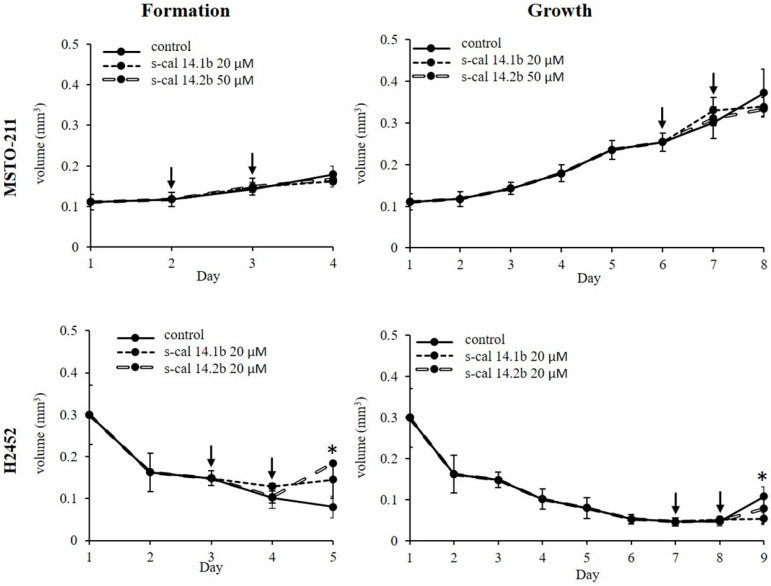
Final volume (mm^3^) of spheroids during formation and growth period; arrows indicate the day in which conotoxins were added to culture. * Statistical difference vs control cultures unexposed; *p* ≤ 0.05.

The total volume of spheroids corresponding to the formation period is shown in Figure 2—Formation. The conotoxins had no significant effect on the final volume in the MSTO-211H cells as compared with non-exposed spheroids (statistical analyses are present in the Supplementary Table S1). During the formation period, in H2452 cells, spheroids showed a major volume after exposure to conotoxins compared to the unexposed (Figure 2—Formation; asterisk), and the final volume reached statistical significance (*p* = 0.028 and <0.001; Supplementary Table S1). Additionally, the effect of conotoxin exposure on total spheroid volume during the growth period was evaluated. The stimulus was applied on days six and seven for MSTO-211H cells and days seven and eight for H2452 cells, and maintained for 24 h more (Figure 2—Growth). In the MSTO-211H spheroids, there were no differences in the total volume. In contrast to the formation period, the volume exhibited by exposed H2452 cultures during the growth period was smaller than that of the control (Figure 2—Growth); the volume of spheroids exposed to s-cal14.1b reached a statistically significant difference (asterisk, *p* = 0.024; Supplementary Table S1).

**Figure 3:**
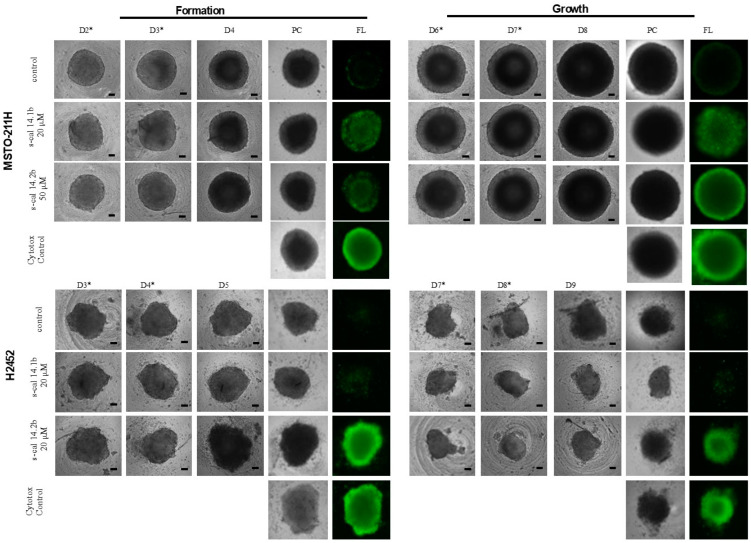
The spheroid morphology during formation and growth was monitored for a long time. * The day conotoxins were added to culture. Panels titled PC correspond to phase contrast and FL to fluorescent mark (green) of cytotoxic effects; all images were taken under the same epifluorescence microscopy parameters. Barr = 100 μm.

The morphologic appearance of 3D cultures during both formation and growth is shown in Figure 3. The MSTO-211H spheroids had a rounder shape since the first day and exhibited no substantial changes in their morphology during both assay periods (Figure 3, MSTO-211H panel). In contrast, H2452 spheroids showed a less round 3D shape and seemed larger and looser during the first five days, but smaller and more compact on day nine as compared to the control (Figure 3, H2452 panel).

### 2.4. Conotoxins Induced Cytotoxicity in Spheroids Culture

The potential cytotoxic effects of conotoxins were evaluated by fluorescence microscopy using a commercial probe (see Section 4). Both conotoxins s-cal14.1b and s-cal14.2b induced a cytotoxic effect on MSTO-211H spheroids in both periods, and the effect was higher during the growth period compared to the formation period; seen as increased uptake of fluorochrome (Figure 3, MSTO-211H- FL panel). In H2452 spheroids, only s-cal14.2b induced a significant cytotoxic effect in both periods, formation and growth (Figure 3, H2452-FL panel).

### 2.5. Differential Protein Expression in Epithelial Cell Lines After Conotoxin Treatment

Protein-expression profiles of multicellular spheroids were analyzed, firstly by the PAGE-SDS conventional technique and then by mass spectrometry. The total protein pattern obtained by PAGE-SDS was analyzed in monolayers and 3D cultures and the total number of bands after each condition was analyzed using the GelAnalyzer2010A software (offline software). The change from monolayer to spheroid culture induced a modification in the pattern of expression observed in PAGE for both cell lines (Supplementary Figure S1A, M vs. US). The protein expression of spheroids exposed to s-cal14.1b and s-cal14.2b conotoxins exhibited a differential expression pattern for each cell line (Supplementary Figure S1A). According to semiquantitative analysis, the protein band number was significantly different between the MSTO-211H monolayer and spheroid unexposed cultures (Table 1, *p* = 0.040 day 4 and *p* = 0.005 day 8). Similarly, only MSTO-211H spheroids exposed to s-cal14.1b showed differences in protein band numbers compared to the corresponding untreated spheroids (Table 1, *p* = 0.041). Regarding protein bands in the H2452 culture, a decrease was observed in growth compared to the formation period; however, none of these differences were statistically significant (Table 1).

### 2.6. Mass Spectrometry Analysis

**Figure 4:**
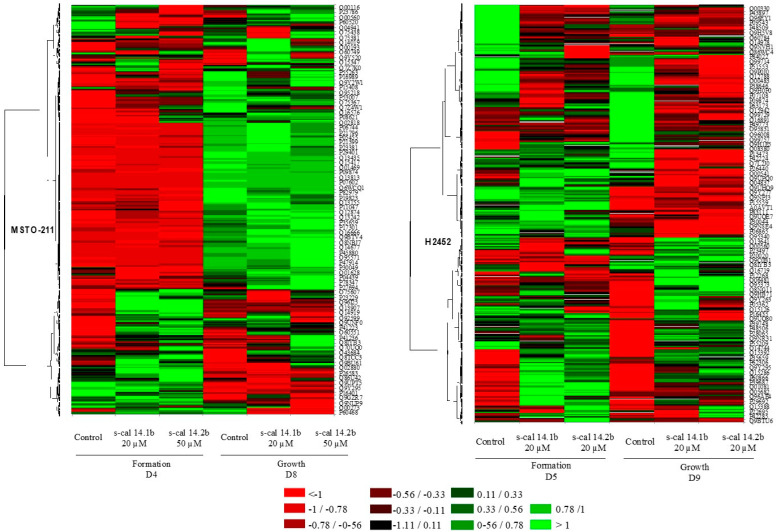
Heat maps representing the differential expression according to experimental condition; control refers to proteins in untreated 3D culture (they were previously compared to 2D culture). All treatments’ expressions were compared to the corresponding 3D control. The expression value rang is indicated by color, green for upregulated and red for downregulated.

To characterize the changes induced by conotoxins in spheroid cultures more extensively, we performed mass spectrometry analyses. For MSTO-211H spheroids, 1635 and 1673 proteins were quantified at day 4 (D4) and day 8 (D8), respectively. In H2452 spheroids, 1522 and 1532 proteins were quantified at D5 at D9, respectively. The mass spectrometry technique exhibited a significantly larger number of proteins for each experimental condition than the PAGE technique. To simplify the analysis, total proteins differentially expressed within a fold change ≥1.5 compared with the untreated conditions were considered for future analyses. In MSTO-211H spheroids, 619 and 646 proteins were found on D4 and D8, respectively, whereas in the H2452 spheroids, 593 proteins were found on D5 and 614 on D9. Although conotoxin exposure did not seem to modify the total number of proteins expressed in both periods for either cell line, a differential expression pattern of spheroids was detected when comparing treated and untreated cells for each cell line (Figure 4). The MSTO-211H spheroids downregulate a larger number of proteins during the formation period, which changed during the growth period, where it seemed upregulated (Figure 4 MSTO-211H, D4 red lines vs D8 green lines). The expression patterns were more heterogeneous in H2452 spheroids, with fewer downregulated proteins in the formation period than in the growth period (Figure 4, H2452 D5 red lines vs D9 green lines). Proteins that were differentially expressed in the cell line, culture period, or the conotoxin treatment were investigated and enlisted according to their cell location, and the percentage change is presented in a graphic form (Supplementary Figure S1B (outer (growth period) vs inner (formation period) ring)). MSTO-211H spheroids exhibited higher changes in the proteome during the transition from formation to the growth period; this was observed in the Golgi apparatus, nucleus, mitochondria, plasma membrane, cytoskeleton, and peroxisome after treatment with s-cal14.1b. In contrast, when MSTO-211H spheroids were treated with s-cal14.2b, more mitochondria, endoplasmic reticulum, and lysosome proteins were found during the transition between periods (Supplementary Figure S1B (outer (growth period) vs. inner (formation period) ring)). H2452 spheroids treated with s-cal14.1b showed an increase in the expression of mitochondria, plasma membrane, cytoplasm, and endoplasmic reticulum proteins, whereas those treated with s-cal14.2b exhibited an increase in nuclei, mitochondria, and cytoskeleton proteins between the formation and growth periods (Supplementary Figure S1B).

#### Protein Expression Induced by s-cal14.2b Generates More Abundant Biological Networks

**Figure 5:**
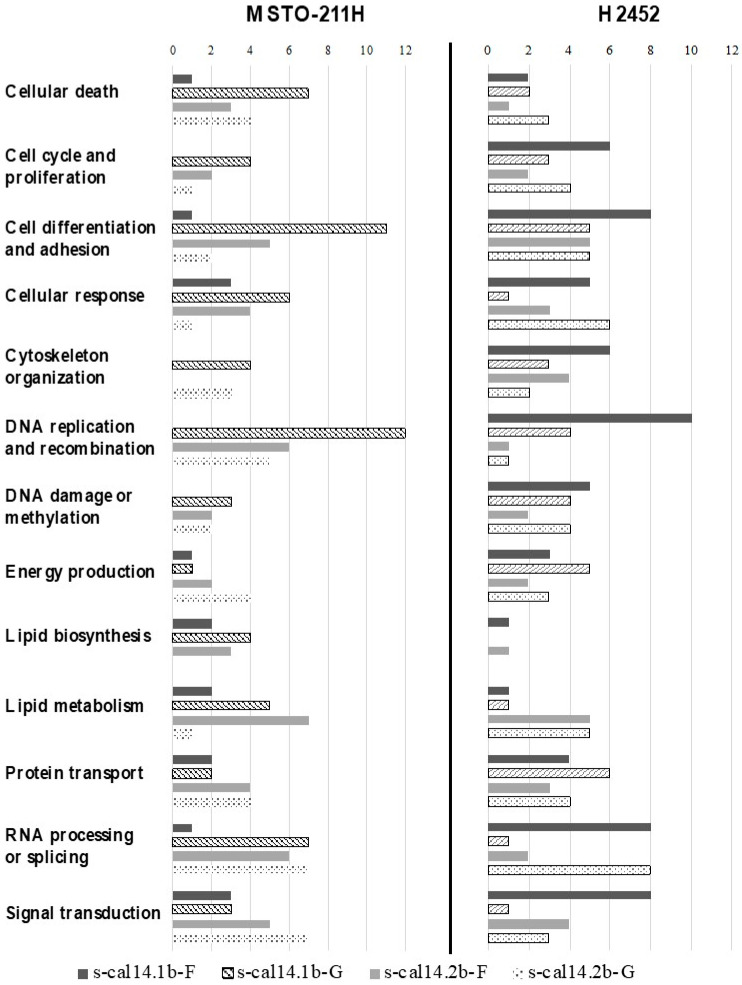
Biological process most significant expressed by 3D cultures according to the experimental condition. Proteins are grouped according to their participation in several processes. F and G, after the toxin’s name, represent the formation or growth period, respectively.

To determine which biological functions could be modified by conotoxin action, we analyzed all differentially expressed proteins. The biological functions in accordance with the UNIPROT database (www.uniprot.org, accessed on 1 June 2021–30 July 2021 [26]) are enlisted in Supplementary Tables S2–S5, and grouped by cell line, conotoxin exposure, and culture period (formation or growth). In MSTO-211H spheroids, s-cal14.1b and s-cal14.2b seemed to have more punctual effects in both studied periods, with less or no change from each other in the number of proteins involved in each biological process analyzed. The most significant changes were observed in cell differentiation and adhesion, DNA replication and recombination for s-cal14.1b, and in the cytoskeleton for both conotoxins during the growth period (Figure 5—MSTO211H).

Similarly, one of the most significant changes observed was cell differentiation and adhesion, and DNA replication and recombination in H2452 spheroids treated with s-cal14.1b. In contrast, RNA processing/splicing was increased by both conotoxin treatments, whereas lipid biosynthesis was modified only during the growth period (Figure 5—H2452).

**Figure 6:**
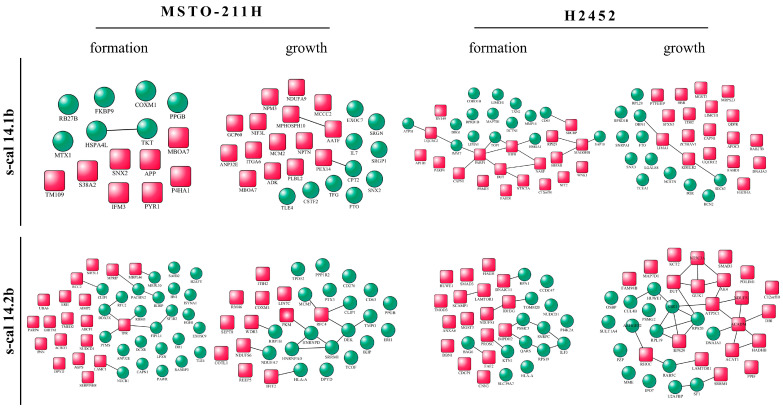
Interaction between proteins in MSTO-211H or H2452 3D cultures. Red squares indicate downregulated proteins and green circles upregulated ones. Interactions between proteins are indicate by black lines. The relationship was generated by Cytoscape software.

The upregulated and downregulated proteins with unique expression under each experimental condition were analyzed to search for direct interactions by means of String v. 12.0. and Cytoscape software v.3.8.0. [27]. In MSTO-211H, spheroids exposed to s-cal14.1b did not show a direct relationship with each other, whereas those exposed to s-cal14.2b showed a greater number of interactions (Figure 6—MSTO-211H). In contrast, upregulated and downregulated proteins in H2452 spheroids exposed to s-cal14.1b during the formation period and s-cal14.2b during the growth period showed an important number of interactions (Figure 6—H2452).

### 2.7. Intravenous Administration of s-cal14.1b Decreased the Tumor Mass of MPM Xenografts

**Figure 7:**
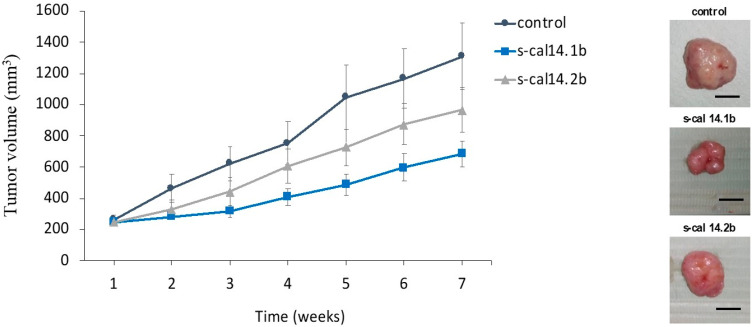
Tumor growth in xenograft model was inhibited by s-cal14.1b; volume is presented as mean ± standard deviation, the follow up was seven weeks. s-cal14.1b showed statistical difference with respect at untreated group mice (blue vs. black line). Right panel, representative images of tumor mass, after sacrifice of the mice. Barr = 15 mm.

Finally, the possible antitumor activity of the conotoxins was studied in a mouse xenograft MPM model. The mice treated with s-cal14.2b exhibited less growth than the control group (untreated mice) (Figure 7, triangles vs. circle symbols). Importantly, the tumor mass of mice treated with s-cal14.1b grew slower compared to the other two groups (Figure 7, square vs. all symbols), from the second week to the end of the study; the final tumor volume was statistically significant with respect to the control (Figure 7, squares vs. circle symbols). Representative images of the tumor mass extracted from one mouse in each group are presented on the right side (Figure 7—images panel).

## 3. Discussion

The marine environment hosts millions of species, of which only approximately 250,000 have been described. This diversity has drawn the attention of research groups investigating natural marine compounds to develop or design drugs for treating different diseases, including cancer. Peptides from several marine sources have shown antiproliferative and antitumor activities; some of them have been tested in human clinical assays [28,29]. In the present study, two peptides from *Californiconus californicus* in their synthetic form, s-cal14.1b and s-cal14.2b, exhibited the capability to modify the cell proliferation, apoptosis, and tumor growth in 2D and 3D cultures of mesothelioma cell lines, and inhibited the mass tumor growth in mice xenografts. Indeed, an important number of proteins in the 3D cultures of MPM cells were differentially expressed after treatment with these two synthetic peptides. The cell lines chosen for this study have characteristics that are representative of two MPM histologies: The H2452 cell line was derived from the epithelial subtype, while the MSTO-211H line was derived from the biphasic subtype. Our study is in concordance with some studies that suggest that marine compounds can kill cancer cells through classical mechanisms, such as apoptosis activation. However, it has been suggested that some anticancer peptides act on cellular membranes; in particular, cationic peptides are attracted to and bind to the surface of tumor cells, inducing their collapse and death [30].

Apoptosis induction and inhibition of proliferation was observed on 2D MPM cell lines, but notably, normal cells did not experience any inhibitory effects, which allows us to suggest its potential use as a human oncological treatment. Both cal14.1b and cal14.2b peptides were first described by Biggs et al. and are part of the peptide superfamily J2; their sequence corresponds to the attack venom of the *C. californicus* [20]. In previous studies, synthetic peptides obtained from this species showed proapoptotic activity in lung cancer cell lines, via activation of caspases, inducing a high Bax/Bcl-2 ratio [22,23].

The use of 3D spheroid models in the solid tumor study has a closer approach to some biological aspects of tumor cells under ex vivo conditions, such as interactions among cells or nutrient concentration gradients [31]. Few studies have been carried out in 3D MPM cultures with a distinctive spheroid morphology, looking for emerging molecular targets and their respective antineoplastic compounds, like this. Moreover, here, cell growth was followed up for 14 days, the longest time in which cultures remained viable, unlike previous studies, where growth was followed for short periods, i.e., 72 h [32,33]. Proteins are necessary to maintain cell structures; thus, we hypothesized that a larger number of protein–protein interactions could be maintained in 3D MPM cultures. In this sense, their expression can vary considerably from 2D cultures.

Furthermore, we analyzed protein expression in 2D and 3D cultures, in which several proteins were differentially expressed in the 3D culture compared to the 2D control. Understanding the cellular and histological implications of such phenomena is beyond the scope of the present study. We focused on proteins differentially expressed in spheroid cultures following conotoxin exposure. Proteomic and fluorescent approaches have demonstrated that conotoxins can induce cytotoxicity in cultures; in particular, the biphasic cell line was more sensitive. Our results are in concordance with another study, wherein a cyclopeptide derived from *D. auricularia* exhibited a cytotoxic effect on tumor cells, leading to apoptosis; the authors suggest that this occurs through prohibitin-1. In addition, keenamide obtained from the marine mollusk P. forskalii induced cytotoxicity in P-388, A549, and HT-29 cell lines, although its molecular targets remain unknown [34,35].

A high diversity of bioactive molecules in venoms was defined a few decades ago, which have been used to design pharmacologically active substances that target ion channels and central nervous system receptors. Thus, their pharmacological activity is ideal as an analgesic, examples of which are ziconotide and tanezumab [14,36,37]. The search for a single molecule or a panel of molecules with biological activity that could be introduced into clinical practice with promising activity to change the treatment landscape in MPM is required. In previous studies, small peptides with low molecular weights have been assumed to have higher cellular mobility, diffusivity, high absorption, and minimal toxicity, which allows them to increase intracellular interactions and improve anticancer activity [38,39]. Currently, nine drugs based on molecules of marine origin have been approved by the FDA and are commercially available, namely cytarabine, trabectedin, eribulin mesylate, brentuximab vedotin, plitidepsin, polatuzumab vedotin, enfortumab vedotin, and belantamab mafodotin; around 20% of the peptides with anticancer activity are derived from the Mollusca phylum [38]. At least three of these commercial drugs showed activity on the cytoskeleton and participated in apoptosis, migration, invasion, and cell cycle regulation [40]; in concordance with this, conotoxins used here modified the expression of most proteins related with such biological functions in our 3D cultures.

The lack of biomarkers or mutations with therapeutic implications has prevented the development of personalized therapies for MPM patients. In addition, the application of functional proteomics in oncological research could allow a better understanding of tumor cell interactions and favor the development of new therapies for MPM. According to our data, both peptides s-cal14.1b and s-cal14.2b can downregulate the expression of proteins involved in cell proliferation, migration, and cell cycle regulation, and promote apoptosis mechanisms; these are vital functions in cancer development. Although complementary functional assays are needed to fully understand the mechanisms, pathways, and targets modified by these peptides, this first approach constitutes a step ahead in the study of the potential anticancer activity of marine peptides.

The emerging mechanisms of resistance to cell death have been recognized as one of the main hallmarks of cancer [41]. In 3D cultures exposed to both conotoxins, we detected the expression of many apoptosis-related proteins including APP, AIMP2, PARP1, PARP4, ANXA6, MCM2, DDX3X, IKBIP, BAG6, SRGN, and PPIF. Most of these have been previously described in several cancer types, including lung, breast, gastric, and ovarian [42,43,44,45]. ANP32E is overexpressed in pancreatic cancer compared with normal tissues, and its silencing inhibits cell proliferation and colony formation in vitro [42]. In addition, it has been indicated as a poor prognostic factor in solid tumors such as pancreatic cancer, triple-negative breast cancer and hepatocarcinoma [42,43,44]. Thus, s-cal14.1b induced ANP32E overexpression. One previous in silico analysis showed that the concurrent expression of ANP32E and CIP2A was a poor prognostic factor in patients with malignant mesothelioma. However, when the analysis included concomitant expression of cyclin B2 and CDK1, it was a good prognostic marker [46]. DDX3 has an oncogenic role in several malignancies and might be a potential target of conotoxins, as it was overexpressed in 3D MPM cultures after conotoxin exposure. In prostate cancer, DDX3 exhibits a differential role according to its subcellular localization, in which the cytoplasmic expression favors higher metastatic activity and progressive disease [47]. In lung cancer, low DDX3 expression is associated with poor prognosis and overall survival [48]. RK33 (diimidazole [4,5-d:40,50-f]-[1,3]diazepine), a synthetic DDX3-specific inhibitor, induced tumor regression in lung cancer mouse models [49], suggesting that this protein might be crucial for cancer treatment. However, their role, expression, and function in MPM remain unclear.

Here, we found an increased expression of Annexin-6 (AnxA6). Although there is no information about its role in MPM, this protein has a controversial role in cancer cell proliferation, invasion, metastasis, apoptosis, and drug resistance depending on cancer type [50]. It is possible to suggest that an increased expression of AnxA6 on the cell membranes might allow greater exposure to the toxin; previous works with marine toxins have shown that some small peptides can induce apoptosis or promote cell cycle arrest in tumor cells, mainly via the activation of caspases or p53 [38,51]. In this regard, our study did not find a specific modification of caspase expression in 3D culture conotoxins post-exposure, although we did find an induction of apoptosis in monolayer cultures. Moreover, conotoxins modify the expression of several proteins associated with apoptotic mechanisms and cell cycle regulation, suggesting alternative pathways that participate in their cytotoxic effect.

PARP1 expression decreased after exposure to conotoxins, which are implicated in the DNA repair pathway. Most of the evidence suggests that PARP inhibition promotes better survival outcomes in some oncological tumor types. PARP inhibition induces cell death via proapoptotic pathways. Nowadays, PARP1 inhibitors alone or in combination, are widely used in clinical practice (reviewed in [52,53]). PARP4, another member of the PARP superfamily, has not been reported in samples of MPM, although it has been reported in breast or thyroid cancer. Unfortunately, these studies have not been conclusive about their role in carcinogenesis [54,55]. Both proteins PARP-1 and -4 could be good targets to test in combination with conotoxins and chemotherapy. Recently, their location in mitochondria has been described, although their precise function remains unknown (reviewed in [56]). Regarding the changes observed in cellular location, mitochondrial proteins exhibited major changes between the formation and growth period of cultures. These changes may be related to the high activity of conotoxins in apoptotic pathways or energy production. Therefore, complementary assays using different approaches are required.

FTO, DEK, HMGA1, RPA1, CUL4B, and HUWE1, which participate in DNA repair, were found overexpressed in our experimental assays. DEK expression may influence the outcome of PARP-inhibitor treatment in cell cultures [57]. DEK overexpression has been correlated with tumor size and stage in pancreatic tumors, as well as with the probability of metastasis [58]. Indeed, DEK overexpression in vitro was associated with an increase in EGFR, KRAS, and vimentin levels in lung cancer cell lines and poor prognosis according to patient database analyses [59]. CUL4B appears to have a similar function in MPM, lung cancer and ovarian cancer, and is correlated with poor prognosis [60,61,62].

Marine peptides have demonstrated encouraging anticancer activity, such as inhibition of cell proliferation in vitro or induction of cell death in several tumor cell lines. Among them, there are hemiasterlins (family of linear peptides derived from sponges) and dolastatins (Dolastatin 10 and Dolastatin 15), which have even arrived at phase II studies. However, these studies were halted based on the high toxicity induced in patients and low in vivo efficacy [39]. In contrast, our study did not find apoptosis in normal cell line (monolayers) or toxicity signals in mice models; this could suggest that the toxin administration would have a lower toxicity induction in human patients.

Two molecular processes that are strongly associated with cancer development are cell cycle and cytoskeleton organization. The expression of M-PRIP, CAPN1, CLIP, COTL1, SEPTIN8, LIMA1, LIMC1, DBN1, and MA7D1, which are involved in cytoskeleton organization or remodeling, as well as TPR, JPT, MCM2, MCM5, SDCBP, LAMTOR1, TMOD3, SDCB1, and CUL4B, all of which are involved in cell cycle, was altered after conotoxin exposure. However, the expression level was different between s-cal14.1b and s-cal14.2b exposure 3D cultures. Thus, further studies on their presumed microtubule and mitosis inhibition might shed light on their mechanisms of action.

No major changes in immune response proteins were observed in MSTO-211H spheroids compared with H2452, including NUDCD1, CD82, CD276, IFM3, HLA-A, IFIT2, DDX3, and BAG6, and the last three are also involved in apoptotic processes. These proteins could be a subject of study based on their role in immune activation and inflammatory regulation in mesothelioma carcinogenesis due to asbestos exposure, the main etiological expositional factor [63].

Importantly, we identified an extensive number of proteins in our samples whose function has not yet been elucidated in MPM, and further analyses are required to establish their roles during its genesis. In previous studies using human samples and mass spectrometry techniques, 52 proteins showed distinctive expression between MPM and benign tissue, which can vary based on the histological subtype [64,65]. Hence, we hypothesized that protein-expression levels varied according to the histological subtype, which has been linked to intrinsic differences in each subtype; the biphasic subtype is more aggressive than the epithelial subtype. Several proteins converge in a unique molecular pathway or interact with many other pathways, suggesting that an ensemble of proteins may serve as markers or therapeutic targets. Additionally, marine molecules can be used to design new biologically active drugs against these proteins for therapeutical purposes.

According to both European and American drug-regulating agencies, the four marine drugs Cytostar-U, Yondelis, Halaven, and Adcetris are listed as anticancer drugs [39]. Moreover, worldwide there are many molecules that are in different phases of studies, and some are even registered in clinical trials. Here, the antitumoral effect of conotoxins was confirmed in a mouse xenograft model, in which s-cal14.1b had a significant inhibitory effect on tumor growth without any toxicity concerns. Furthermore, we highlight that both synthetic conotoxins decreased the final volume of the tumor; this result was in concordance with the cytotoxicity observed in 3D cultures. However, based on those observed in the heatmap, interaction networks, and morphological ubication changes of proteins modified by each conotoxin, it is possible to suggest that each has a specific mechanism of action. Despite this, we observed that such modifications could converge in similar biological functions. Studies aimed at discerning the mechanism of action of each of the conotoxins inside tumor cells, mainly in the 3D culture models and xenografts, are necessary to elucidate the role of s-cal14.1b or s-cal14.2b as potential anticancer agents. Finally, it should be considered that the mode of action of each conotoxin could be modified through intracellular or environmental interactions in both spheroids and xenograft models.

Finally, we want to mention that although here we did not find toxicity induction in normal cells or mice, this does not necessarily mean that these are safe for humans; this could be considered a limitation of our study. Additionally, a comparison of the antitumor effects of conotoxins vs one chemo drug used as mesothelioma treatment was not considered; moreover, lack of information about the specific conotoxins for mesothelioma cells could be a limitation of this study.

In summary, s-cal14.1b and s-cal14.2b peptides reduced in vitro cell proliferation and induced apoptosis and cytotoxicity. Here, were identified 3D culture-specific proteins with differential expression in response to conotoxin exposure. Moreover, we demonstrated an antitumor effect in xenografted mice treated with conotoxins. One of the most important objectives of this work was to propose a set of proteins that could function as cellular targets for such peptides, which would allow them to be used as therapeutic agents. Here, we suggest that further research work with peptides could assess the role of AATF, PARP1, PARP4, HUWE1, APP, CAPN1, LIMA1, or ANXA6 proteins, based on their change in expression post-treatment with conotoxins, their involvement and relationship in apoptosis, cell adhesion and differentiation, and cytoskeleton modification (Supplementary Figure S2). Ideally, the mechanisms of action and interactions between conotoxins and tumor cells should be explored and subsequently scale the study toward more robust models and to clinical trials.

## 4. Materials and Methods

All materials and reagents were purchased from Sigma-Aldrich (St. Louis, MO, USA), unless otherwise indicated.

### 4.1. Cell Culture

The cell lines MSTO-211H (CRL-2081, ATCC, Manassas, VA, USA) and H2452 (CRL-5946, ATCC, Manassas, VA, USA), derived from MPM, and the normal lung tissue-derived cell line MRC-5 (CCL-171, ATCC, Manassas, VA, USA) were maintained in RPMI-1640 high-glucose medium and minimum essential medium (MEM) (GIBCO, Thermo Fisher Scientific, Bohemia, New York, NY, USA), respectively, supplemented with 10% fetal bovine serum (FBS, ATCC, Manassas, VA, USA). All the cell lines were incubated in a 5% CO_2_ atmosphere at 37 °C.

### 4.2. Conotoxins s-cal14.1b and s-cal14.2b

Both conotoxins cal14.1b (GDCPPWCVGARCRAGKC) and cal14.2b (RECPPRCP TSHCNAGTC) were first identified from a cDNA library from *Californiconus californicus*, named and reported by Biggs et al., 2010 [21]. In the present study, we used the synthetic forms (s-cal14.1b and s-cal14.2b) obtained from Agentide Inc. (Morristown, NJ, USA).

### 4.3. Animal Model

Male athymic nu-/nu- mice of six weeks of age were obtained from the Unidad de Producción y Experimentación de Animales de Laboratorio (UPEAL-CINVESTAV Zacatenco, Mexico City, Mexico). Animals were kept in a pathogen-free environment in a micro-isolator and fed autoclaved food and water ad libitum. All applicable institutional and governmental regulations were followed in agreement with the Mexican Federal Regulations for Animal Production, Care, and Experimentation (NOM-062-ZOO-1999, Ministry of Agriculture; Mexico City, Mexico). Guidelines from the Guide for Care and Use of Laboratory Animals of the National Institutes of Health (https://www.ncbi.nlm.nih.gov/books/NBK54050/, accessed on 15 October 2024, doi: 10.17226/12910) were also followed. All efforts were made to minimize suffering and to reduce the number of animals used. This protocol was approved by both Ethical and Investigation Committees (018/052/IBI) (CEI/1292/18).

### 4.4. Cellular Proliferation and Apoptosis Assays in 2D Cultures

The cell lines were seeded at 3 × 10^3^ cells/well and maintained for 24 h in complemented culture media, followed by exposure to increasing concentrations of each toxin (20, 30, and 50 μM) for 24 and 48 h. Proliferation was measured using crystal violet at the end of the conotoxin exposure. The samples were washed with 1× PBS and fixed with 3.7% formaldehyde. The samples were then stained with 0.1% crystal violet overnight, and 33% acetic acid was added to the samples and the absorbance was read at λ = 570 nm in a spectrophotometer (model Stat Fax 4200, Awareness Technology Inc., Palm City, FL, USA). For the apoptosis assay, conotoxins at final concentrations of 20 and 50 μM were used, and the samples were stained with an annexin-propidium iodide kit, according to the manufacturer’s instructions (Roche, cat. 11988549001), and read using an Attune Nxt (Thermo, Bohemia, New York, NY, USA) flow cytometer model. Additionally, 0.5 μM staurosporine (final concentration) was used as the positive internal control. All samples were made independently in triplicate.

### 4.5. Formation and Growth of Multicellular Spheroids

For the multicellular spheroids in the plate, 10 × 10^3^ and 5 × 10^3^ cells/well of H2452 and MSTO-211H cells, respectively, were seeded in 96 wells for the 3D plate (Corning, New York, NY, USA). The cells were maintained in 4% FBS and 0.2% Penicillin-Streptomycin in a CO_2_ atmosphere at 37 °C. Spheroid growth was followed for 14 days, and the medium size was calculated through the daily photographic record with an optical microscope Axio Observer A.1. (20x/1.1 objective, Carl Zeiss, Mexico City, Mexico) and was measured using Axio Vision software v.4.9.1 (Carl Zeiss, Mexico). The spheroid volume was calculated using the equation V = (Dxd^2^)(π/6), where D is the larger diameter and d is the short diameter. All experiments were performed in triplicate in three independent assays.

### 4.6. Exposure of Multicellular Spheroids to Conotoxins

The 3D cell cultures were exposed to s-cal14.1b and s-cal14.2b conotoxins at a final concentration of 20 μM for H2452 and 20 μM (s-cal14.1b) and 50 μM (s-cal14.2b) for MSTO-211H cells. Subsequently, protein expression was analyzed under these conditions.

The multicellular spheroids were exposed to conotoxins, at two and three formation days for MSTO-211H cells and three and four formation days for H2452 cells. For growth assays, the conotoxins were added to cell cultures at seven and eight days for the MSTO-211H cell line, and eight and nine days for the H2452 cell line. The stimuli were maintained for 24 h more after each period, and the total volume of spheroids corresponding was determined.

### 4.7. Cytotoxicity Assay on Multicellular Spheroids

Spheroids grown under normal culture conditions (5% CO_2_ at 37 °C) were exposed to conotoxins, as described in the previous section. At the end of the treatment, the CellTox^TM^ Green Cytotoxicity Assay Kit (G8741, Promega, Madison, WI, USA) was used according to the manufacturer’s instructions. Samples were then analyzed by mean fluorescence microscopy using an Axio Observer A.1 inverted microscope (Carl Zeiss, Mexico City, Mexico) for image analysis, and approximately 500 total cells were counted with an objective plan-neofluar 20X0.50, and images were taken with ZEN v 2.3 software (Carl Zeiss, Mexico City, Mexico).

### 4.8. Protein Extract and Denaturing Gel Electrophoresis Assays

The samples were prepared according to the spheroid formation and growth time. Briefly, spheroids treated with or without each toxin were washed out with 1× PBS, and resuspended in lysis and extraction buffer RIPA complemented with a protease inhibitor cocktail (Complete EDTA-free (Roche, Indianapolis, IN, USA) and sodium orthovanadate). Samples were centrifuged at 14,000 rpm, the supernatant was recovered, and the total protein content was quantified by the Bradford method using a NanoDrop2000 spectrophotometer (Thermo Fisher Scientific, Bohemia, New York, NY, USA). Additionally, the total protein content from monolayer cultures of MSTO-211H, H2452, and MRC-5 cells was obtained in the same manner as for spheroids. SDS-PAGE was performed on a 10% polyacrylamide gel. Twenty micrograms of purified total protein extract of each sample were loaded onto the gel and stained with silver nitrate, and digital images were captured using Image J Java 1.8.0-112 and GelAnalyzer 2010a software (available at www.gelanalyzer.com, accessed on 15 October 2024).

### 4.9. Protein Digestion and Mass Spectrometry Analysis

The Pierce Mass Spec Sample Prep Kit for cultured cells (cat. 84840, Thermo Fisher Scientific, Bohemia, New York, NY, USA) was used for mass spectrometry analysis following the manufacturer’s instructions. Briefly, approximately 120 μg of total protein in lysis buffer was resuspended in 0.1% formic acid and analyzed using an Eksigent NanoLC 400 high-performance liquid chromatographer (AB SCIEX, Dublin, CA, USA) coupled to a TripleTOF 5600+ mass spectrometer (MA, AB SCIEX, Framingham, MA, USA) equipped with a DuoSpray ion source.

#### 4.9.1. Protein Identification/Proteomic Processing Data

The spectra data obtained directly from the MS were processed by software ProteinPilot 4.5 (AB Sciex) using the Paragon algorithm to search for matches within the UniProtKB human (50, 351 entries)-curated database [25]. Trypsin was considered as the digestion enzyme, iodoacetamide, a cysteine alkylating agent, and identification was established at >95% probability.

#### 4.9.2. Heat Maps and Visualization Analysis

Differential protein expression according to each experimental condition was graphed as a heat map using the XLSTAT software v2020.1.1.64431 (Addinsoft, Paris, France). We filtered the protein expression using only those with a fold change ≥1.5 compared with control cells (untreated). The inter-relationship was observed in the protein database and expressed as a solid line; all those without interplay are not indicated.

### 4.10. Mice Malignant Pleural Mesothelioma Xenografts

Athymic mice were inoculated with 3 × 10^6^ MSTO-211H cells/100 μL of culture medium on the dorsal flank; five mice were included in each experimental group. The tumor mass growth was monitored on days with a gauge, starting two weeks after the initial inoculation, then tumor volume was obtained using the equation V = (Dxd^2^)(π/6), where “D” is the larger diameter and “d” is the short diameter. When the tumors reached approximately 200 mm^3^, the conotoxins were dosed once a week with five total doses, followed by one extra week. s-cal14.1b and s-cal14.2b were intravenously administered to mice in injectable water in the caudal vein at a final concentration of 0.5 mg/kg. When the treatment ended, the mice were euthanized using isoflurane (2% in 100% oxygen) and the tumor mass was obtained. Tumoral growth is presented as means (mm^3^) ± SD (standard deviation).

### 4.11. Statistical Analysis

Continuous variables are summarized as arithmetic means and standard deviations. Statistical analysis was performed by two-way analysis of variance (ANOVA) using Tukey’s test. Statistical significance was determined at a *p*-value of 0.05, using SPSS software v20 (SPSS Inc., Chicago, IL, USA). All experimental assays were performed in independent form in triplicate at least three times.

## 5. Conclusions

In conclusion, our work demonstrated the antitumoregenic properties of two synthetic peptides (s-cal14.1b and s-cal14.2b) isolated from venom of *C. californicus*, as they were assayed on a malignant pleural mesothelioma model (in vitro and in vivo). As far as we know, this is the first report on possible cell targets of antitumoregenic synthetic peptides on MPM investigated using proteomic tools.

## Figures and Tables

**Table 1:** Quantification of protein bands in spheroid cultures.

	MSTO-211				H2452			
Condition	Day 4 Mean ± SD	*p*-Value	Day 8 Mean ± SD	*p*-Value	Day 5 Mean ± SD	*p*-Value	Day 9 Mean ± SD	*p*-Value
Monolayer	54 ± 4.1				46 ± 3.6			
Spheroids untreated	41 ± 3.5	**0.040**	29 ± 2.7	**0.005**	51 ± 2.8	0.138	38 ± 3	0.069
s-cal14.1b ^ϕ^	44 ± 3.8	0.366	34 ± 1.3	**0.041**	45 ± 4.9	0.251	38 ± 2.5	0.742
s-cal14.2b ^ϕ^	37 ± 2.4	0.238	33 ± 2.7	0.123	44 ± 3.9	0.112	34 ± 1.1	0.109

^ϕ^ spheroids treated with each conotoxin; SD, standard deviation.

## Data Availability

All data generated or analyzed during this study are included in this published article and its supplementary information files. The raw data supporting the conclusions of this article will be made available by the authors, without undue reservation.

## Supplementary Materials

The following supporting information can be downloaded at https://www.mdpi.com/article/10.3390/md23010032/s1, Figure S1: Electrophoretic profile of proteins expressed in mesothelioma cultures; Figure S2: Interaction between key proteins involved in cellular death, and/or differentiation and cytoskeleton modification; Table S1: Final volume of multicellular spheroids in both periods; Table S2: Proteins modified by conotoxins during formation time of MSTO-211H spheroids; Table S3: Proteins modified by conotoxins during growth time of MSTO-211H spheroids; Table S4: Proteins modified by conotoxins during formation time of H2452 spheroids; Table S5: Proteins modified by conotoxins during growth time of H2452 spheroids.
