# Supplementary material for: Preclinical Efficacy and Proteomic Prediction of Molecular Targets for s-cal14.1b and s-cal14.2b Conotoxins with Antitumor Capacity in Xenografts of Malignant Pleural Mesothelioma
Source: Mar Drugs. 2025 Jan 10;23(1):32. doi: 10.3390/md23010032 (PMC11767107; doi:10.3390/md23010032)
Supplement: Supplementary file 1 [file marinedrugs-23-00032-s001.zip › marinedrugs-3281450-supplementary/Table S1.pdf]

Table S1. Final volume of multicellular spheroids in both periods.										
Period	Formation					Growth				
Condition / cells	ctl	s-cal14.1b	p-value	s-cal14.2b	p-value	ctl	s-cal14.1b	p-value	s-cal14.2b	p-value
MSTO-211H	0.179 ± 0.020	0.162 ± 0.014	0.178	0.167 ± 0.011	0.331	0.372 ± 0.057	0.339 ± 0.022	0.398	0.333 ± 0.004	0.305
H2452	0.090 ± 0.019	0.145 ± 0.044	**0.028***	0.184 ± 0.006	**<0.001***	0.104 ± 0.019	0.054 ± 0.015	**0.024***	0.079 ± 0.036	0.341

* Statistical significance compared to untreated spheroids at same day; t-student, Tukey
