# Supplementary material for: Preclinical Efficacy and Proteomic Prediction of Molecular Targets for s-cal14.1b and s-cal14.2b Conotoxins with Antitumor Capacity in Xenografts of Malignant Pleural Mesothelioma
Source: Mar Drugs. 2025 Jan 10;23(1):32. doi: 10.3390/md23010032 (PMC11767107; doi:10.3390/md23010032)
Supplement: Supplementary file 1 [file marinedrugs-23-00032-s001.zip › marinedrugs-3281450-supplementary/Table S2.pdf]

Table S2. Proteins modified by conotoxins during formation time of MSTO-211H spheroids.

Conotoxin	ID protein	Abreviattion	Name	Biological process
**s-cal14.1b**				
	P05067	APP	Amyloid Precursor Protein	Apoptosis, Cell adhesion, Endocytosis, Notch signaling pathway
	O60749	SNX2	Sorting nexin-2	Protein transport, Endocytosis
	Q96QD8	S38A2	Sodium-coupled neutral amino acid transporter 2	Aminoacid transport, Cellular response
	Q96N66	MBOA7/LPCAT1	Lysophospholipid acyltransferase 7	Lipid biosynthesis and metabolism
	Q9BVC6	TM109	Transmembrane protein 109	Ion transport, Cellular response
	Q01628	IFM3	Interferon-induced transmembrane protein 3	Immune response
	P27708	PYR1	CAD protein	Pyrimidine pathway biosynthesis
	P13674	P4HA1	Prolyl 4-hydroxylase subunit alpha-1	Oxidoreductase activity
	O00194	RB27B	Ras-related protein Rab-27B	GTPase activity
	Q7Z7K0	COXM1	COX assembly mitochondrial protein homolog	Undefined
	P29401	TKT	Transketolase	Transketolase activity, Calcium ion binding

	O95757	HS74L	Heat shock 70 kDa protein 4L	ATPase, Protein folding, Stress response
	P10619	PPGB	Lysosomal protective protein (cathepsin A)	Proteolysis, Protein stability and transport
	Q13505-2	MTX1	Metaxin-1	Mitochondrion organization, Protein transport
	O95302	FKBP9	Peptidyl-prolyl cis-trans isomerase FKBP9	Protein folding
**s-cal14.2b**	Q9NY61	AATF	Protein AATF	Cellular response, Cell adhesion, Transcription, Cell cycle
	Q9BTT0	ANP32E	Acidic leucine-rich nuclear phosphoprotein 32 family member E	Apoptosis, Chromatin regulator
	P23229	ITGA6	Integrin alpha-6	Cell-cell adhesion, Cellular response, Cell migration
	Q9HCC0	MCCC2/MCCase	Methylcrotonoyl-CoA carboxylase beta chain, mitochondrial	Metabolic process
	P49736	MCM2	DNA replication licensing factor MCM2	Cell cycle, DNA replication, Apoptosis, Cellular response
	O00566	MPHOSPH10	U3 small nucleolar ribonucleoprotein protein MPP10	rRNA processing, RNA splicing
	Q16795	NDUFA9	NADH dehydrogenase [ubiquinone] 1 alpha subcomplex subunit 9, mitochondrial	Electron transport, Energy production
	O75607	NPM3	Necleoplasmin-3	Chromatin remodeling, rRNA processing, rRNA transcription

	Q9Y639	NPTN	Neuroplastin	Cell adhesion, Neurogenesis
	O75381	PEX14	Peroxisomal membrane protein PEX14	Protein transport and ubiquitination, DNA transcription
	Q8NHP8	PLBL2	Putative phospholipase B-like 2	Lipid metabolism and degradation,
	Q96N66	MBOA7/LPLAT 7	Lysophospholipid acyltransferase 7	Lipid biosynthesis and metabolism
	Q9GZT8	NIF3L	NIF3-like protein 1	mRNA processing, splicing and transport
	P55263	ADK	Adenosine kinase	Energy production
	Q9H3P7	ACBD3/GCP60	Golgi resident protein GCP60	Lipid metabolism and biosynthesis, Steroid synthesis
	O60749	SNX2	Sorting nexin-2	Protein transport, Endocytosis
	Q92734	TFG	Protein TFG	ER-Golgi transport
	Q04727-2	TLE4	Transducin-like enhancer protein 4	Transcription, Cellular response
	P23786	CPT2	Carnitine O-palmitoyltransferaxse 2, mitochondrial	Lipid metabolism and transport
	P13232	IL7/IL-7	Interleukin-7	Signal transduction, Immune response, Cell signaling

	P33240	CSTF2	Cleavage stimulation factor subunit 2	mRNA processing
	Q9C0B1	FTO	Alpha-ketoglutarate-dependent dioxygenase FTO	DNA repair and demethylation, RNA repair
	Q9UPT5	EXOC7	Exocyst complex component 7	Exocytosis, Protein transport
	P10124	SRGN	Serglycin	Apoptosis, Biomineralization, Proten procesing
	Q7Z6B7	SRGAP1/SRGP1	SLIT-ROBO Rho GTPase-activating protein 1	Cell migration, Signal transduction
Shaded cells indicate subexpressed proteins; all functions indicated in biological process were obtained from uniprotkB protein data base				

REF 28. UniProt Consortium. UniProt: the universal protein knowledgebase in 2021. Nucleic Acids Res. 2021 Jan 8;49(D1): D480-D489. doi: 10.1093/nar/gkaa1100. PMID: 33237286; PMCID: PMC7778908.
