# Supplementary material for: Preclinical Efficacy and Proteomic Prediction of Molecular Targets for s-cal14.1b and s-cal14.2b Conotoxins with Antitumor Capacity in Xenografts of Malignant Pleural Mesothelioma
Source: Mar Drugs. 2025 Jan 10;23(1):32. doi: 10.3390/md23010032 (PMC11767107; doi:10.3390/md23010032)
Supplement: Supplementary file 1 [file marinedrugs-23-00032-s001.zip › marinedrugs-3281450-supplementary/Table S3.pdf]

Table S3. Proteins modified by conotoxins during growth time of MSTO-211H spheroids.

Conotoxin	ID protein	Abreviattion	Name	Biological process
**s-cal14.1b**				
	Q8NE71	ABCF1/	ATP-binding cassette sub-family F member 1	Transmembrane transport, Translation, Inflammatory response
	Q9H3P7	ACBD3/GCP60	Golgi resident protein GCP60	Lipid metabolism and biosynthesis, Steroid synthesis
	O00116	AGPS/Alkyl-DHAP	Alkyldihydroxyacetonephosphate synthase, peroxisomal	Lipid metabolism and biosynthesis
	Q13155	AIMP2	Aminoacyl tRNA synthase complex-interacting multifunctional protein 2	Apoptosis, Cell differentiation, Protein biosynthesis
	Q12882	DPYD/DPD or DHPDHase	Dyhydropyrimidine dehydrogenase [NADP (+)]	Nucleotide metabolism
	Q8IV48	ERI1/HEXO	3´-5´exoribonuclease 1	DNA catabolic process, RNA procesing
	Q9H3K2	GHITM/MICS1	Growth hormone-inducible transmembrane protein	Apoptosis
	P11047	LAMC1/S-LAM	Laminin subunit gamma-1	Cell adhesion and migration
	Q6WCQ1-3	MPRIP/M-RIP	Myosin phosphatase Rho-interacting protein	Citoskeleton organization
	Q9H2W6	MRPL46/MRP-L46	39s ribosomal protein L46, mitochondrial	Mitochondrial biogenesis
	Q9GZT8	NIF3L1	NIF3-like protein 1	DNA transcription

	Q96RS6	NUDCD1	NudC domain-containing protein 1	Immune response
	Q9UKK3	PARP4/ARTD4	Protein mono-ADP-ribosyltransferase PARP4	Cell death, DNA repair, Inflammatory response
	Q9H307	PNN/DRS	Pinin	Cell adhesion and transcription
	Q9P258	RCC2	Protein RCC2	Cell cycle, migration and adhesion
	P50452	SERPINB8	Serpin B8	Cell-cell adhesion
	Q15363	TMED2	Transmembrane emp24 domain-containing protein 2	Protein transport, Regulator of gene expression
	P12270	TPR	Nucleoprotein TRP	Cell division, Cellular response, Protein transport, Cell cycle, RNA transcription regulator
	A0AVT1	UBA6	Ubiquitin-like modifier-activating enzyme 6	Cellular response, Protein modification
	Q9BTT0	ANP32E	Acidic leucine-rich nuclear phosphoprotein 32 family member E	Apoptosis, Chromatin regulator
	P07384	CAPN1/CANP1	Calpain-1 catalytic subunit	Proteolysis, Mammary gland involution, Signal transduction, Cytoskeleton remodeling
	P30622-2	CLIP1	CAP-Gly domain-containing linker protein 1	Citoskeleton organization, Intracellular transport

	Q7Z4W1	DCXR/XR	L-xylulose reductase	Cellular metabolism
	O00571	DDX3X	ATP-dependent RNA helicase DDX3X	Apoptosis, Transcription, Lipid homeostasis, Immune response, Cell differentiation and response
	Q01658	DR1	Protein Dr1	Transcription
	Q06265	EXOSC9	Exosome complex component RRP45	rRNA processing
	Q6UN15	FIP1L1/hFip1	Pre-mRNA 3´-end-processing factor FIP1	mRNA processing
	O75367	H2AFY/Histone macroH2A1	Core histone macro-H2A.1	Lipid metabolism, Stress response, DNA regulator
	Q9UK76	HN1/JPT1	Jupiter microtubule associated homolog 1	Cell cycle and adhesion
	Q70UQ0	IKBIP/I kappa-B	Inhibitor of nuclear factor kappa-B kinase-interacting protein	Apoptosis
	Q9NPH2	ISYNA1/IPS 1	Inositol-3-phosphate synthase 1	Phospholipid biiosynthesis, Lipid metabolism
	O60711	LPXN	Leupaxin	Cell adhesion, Transcription, Cell migration
	Q8TCC3	MRPL30/L30mt	39s ribosomal protein L30, mitochondrial	Protein transcription

	Q02818	NUCB1	Nucleobindin-1	Protein modification, Metabolism
	Q9UNF0	PACSIN2/SdpII	Protein kinase C and casein kinase substrate in neurons protein 2	Cytoskeleton organization, Endocytosis
	Q96IZ0	PAWR/Par-4	PRKC apoptosis WT1 regulator protein	Apoptosis, Transcription
	P20962	PTMS	Parathymosin	Immune response
	Q9H6Z4	RANBP3/RanBP3	Ran-binding protein 3	Protein transcription
	P98179	RBM3	RNA-binding protein 3	RNA regulation, RNA processing, Stress response
	P35250	RFC2	Replication factor C subunit 2	DNA damage response, DNA replication
	Q14151	SAFB2/SAF-B2	Scaffold attachment factor B2	mRNA processing, Transcription
	Q13435	SF3B2	Splicing factor 3B subunit 2	mRNA processing, RNA splicing
	Q04727-2	TLE4	Transducin-like enhancer protein 4	Transcription, Cellular response
	Q9BTY7	HGH1	Protein HGH1 homolog	Unidentified
**s-cal14.2b**				
	P14618	PKM/KPYM	Pyruvate kinase PKM	Translation, Energy production, Apoptosis

	Q14019	COTL1	Coactosin-like protein	Cytoskeleton organization
	Q00765	REEP5	Receptor expression-enhancing protein 5	Transport, ER organization
	P19823	ITIH2	Inter-alpha-trypsin inhibitor heavy chain H2	Metabolism, Protein modification
	Q9H2W6	MRPL46/RM46	39s ribosomal protein L46, mitochondrial	RNA translation
	Q7Z7K0	CMC1/COXM1	COX assembly mitochondrial protein homolog	Undefined
	P09913	IFIT2	Interferon-induced protein with tetratricopeptide repeats 2	Apoptosis, Immune response
	O75380	NDUFS6/NDUS6	NADH dehydrogenase [ubiquinine] iron-sulfur protein 6, mitochondrial	Energy production, Transport
	P35249	RFC4	Replication factor C subunit 4	DNA replication, DNA repair
	Q9UNX4	WDR3	WD repeat-containing protein 3	RNA binding, rRNA processing
	Q92599	SEPTIN8/SEPT8	Septin-8	Cytoskeleton modification, Transport
	Q9NUP9	LIN7C	Protein lin-7 homolog C	Exocytosis, Protein transport
	P26022	PTX3	Pentranxin-related protein PTX3	Immune response
	P08962	CD63	CD63 antigen	Cellular signaling, Protein transport, Cell adhesion and migration
	Q13428	TCOF1/TCOF	Treacle protein	RNA translation

	Q70UQ0	IKBIP/IKIP	Inhibitor of nuclear factor kappa-B kinase-interacting protein	Undefined
	Q14103	HNRNPD/HNRPD	Heterogeneous nucleaer ribonucleoprotein D0	Cellular response, mRNA transcription
	P35659	DEK	Protein DEK	Chromatin organization, DNA repair
	P10619	CTSA/PPGB	Lysosomal protective protein	Proteolysis, Regulation of protein stability, Protein transport
	P33992	MCM5	DNA replication licensing factor MCM5	Cell cycle, DNA replication
	O95182	NDUFA7/NDUA7	NADH dehydrogenase [ubiquinone] 1 alpha subcomplex subunit 7	Energy production, Electron transport, Transport
	P04439	HLA-A	HLA class I histocompatibility antigen, A alpha chain	Immune response
	P30622-2	CLIP1	CAP-Gly domain-containing linker protein 1	Cytoskeleton organization, Intracellular transport
	P42166	TMPO	Lamina-associated polypeptide 2, isoform alpha	Transcription
	Q8IYB3	SRRM1	Serine/arginine repetitive matrix protein 1	mRNA processing and splicing
	Q5ZPR3-4	CD276	CD276 antigen	Immune response
	Q8IV48	ERI1/HEXO	3´-5´exoribonuclease 1	DNA catabolic process, RNA procesing
	P41236	PPP1R2/IPP-2	Protein phosphatase inhibitor 2	Carbohydrate metabolism, Glycogen metabolism

	Q12882	DPYD/DPD or DHPDHase	Dyhydropyrimidine dehydrogenase [NADP (+)]	NADP binding
	Q13151	HNRNPA0	Heterogeneous nucleaer ribonucleoprotein A0	RNA metabolism, mRNA processing and splicing
	P55327	TPD52	Tumor protein D52	Undefined
	Q14684	RRP1B	Ribosomal RNA processing protein 1 homolog B	Apoptosis, Transcription, mRNA processing and splicing
Shaded cells indicate subexpressed proteins; all functions indicated in biological process were obtained from uniprotkB protein data base.				

REF 28. UniProt Consortium. UniProt: the universal protein knowledgebase in 2021. Nucleic Acids Res. 2021 Jan 8;49(D1): D480-D489. doi: 10.1093/nar/gkaa1100. PMID: 33237286; PMCID: PMC7778908.
