# Supplementary material for: Preclinical Efficacy and Proteomic Prediction of Molecular Targets for s-cal14.1b and s-cal14.2b Conotoxins with Antitumor Capacity in Xenografts of Malignant Pleural Mesothelioma
Source: Mar Drugs. 2025 Jan 10;23(1):32. doi: 10.3390/md23010032 (PMC11767107; doi:10.3390/md23010032)
Supplement: Supplementary file 1 [file marinedrugs-23-00032-s001.zip › marinedrugs-3281450-supplementary/Table S4.pdf]

Table S4. Proteins modified by conotoxins during formation time of H2452 spheroids.				
**Conotoxin**	**ID protein**	**Abreviattion**	**Name**	**Biological process**
**s-cal14.1b**				
	Q10567	AP1B1	AP-1 complex subunit beta-1	Protein transport
	Q9HB07	C12orf10/MYG1	MYG1 exonuclease	Cellular exploratory moving
	P07384	CAPN1/CANP1	Calpain-1 catalytic subunit	Proteolysis, Mammary gland involution, Signal trasduction, Cytoskeleton remodeling
	P33316	DUT	Deoxyuridine 5´-triphosphate nucleotidohydrolase mitochondrial	Nucleotide metabolism
	Q6P587	FAHD1	Acylpyruvase FAHD1, mitochondrial	Cellular metabolism
	P07305	H1F0/H10	Histone H1.0	Transcription
	Q96A72	MAGOHB/MGN2	Protein mago nashi homolog 2	mRNA processing, transport and splicing
	P49321	NASP	Nuclear autoantigenic sperm protein	Cell cycle, DNA replication, Protein transport
	Q9NQR4	NIT2	Omega-amidase NIT2	Metabolic process
	Q9H0P0	NT5C3A/5NT3A	Cytosolic 5´-nucleotidase 3A	Nucleotide metabolism
	P09874	PARP1	Poly [ADP-robose] polymerase 1	DNA repair, Apoptosis, Transcription, DNA damage, Cellular response

	Q9UKK3	PARP4/ARTD4	Protein mono-ADP-ribosyltransferase PARP4	Cell death, DNA repair, Inflamatory response
	O14744	PRMT5/ANM5	Protein arginine N-methyltransferase 5	Transcription, DNA methylation, Cellular differenciation
	P62273	RPS29/RS29	40s ribosomal protein S29	mRNA processing and splicing
	O00560	SDCBP/SDCB1	Syntenin-1	Cytoskeleton organization, Cell growth and migration, Cell cycle
	Q8IYB3	SRRM1	Serine/arginine repetitive matrix protein 1	mRNA processing and splicing
	P22695	UQCRC2/QCR2	Cytochrome b-c1 complex subunit 2, mitochondrial	Energy production, Electron transport, Proteolysis, Response to drugs
	Q9H4A3-2	WNK1	Serine/treonine-protein kinase WNK1	Protein transport, Intracellular signal transduction
	Q8NC42	RNF149/RN149	E3 ubiquitin-protein ligase RNF149	Cellular response
	P18859	ATP5PF/ATP5J	ATP synthase-coupling factor 6, mitochondrial	Energy production, Transport
	P08962	CD63	CD63 antigen	Cellular signaling, Protein transport, Cell adhesion, Cell migration
	Q9BR76	CORO1B/COR1B	Coronin-1B	Cytoskeleton organization, Cellular response

	Q14203	DCTN1	Dynactin subunit 1	Cell cycle, division and transport
	Q9Y295	DRG1	Developmentally-regulated GTP-binding protein 1	Transcription
	P17096	HMGA1	High mobility grupoi protein HMG-I/HMG-Y	Transcription, DNA repair
	Q16891-2	IMMT/MIC60	MICOS complex subunit MIC60	Mitocondrial calcium ion homeostasis
	Q9UHB6	LIMA1	LIM domain and actin-binding protein 1	Lipid metabolism, Steroid metabolism, Cell migration, Cytoskeleton modeling
	Q9UPQ0	LIMCH1/LIMC1	LIM and calponin homology domains-containing protein 1	Cytoskeleton organization, Cellular migration
	Q3KQU3-4	MAP7D1/MA7D1	MAP7 domain-containing protein 1	Cytoskeleton organization
	P50281	MMP14	Matrix metalloproteinase-14	Angiogenesis, Lung development, Cell motility
	Q9NQG5	RPRD1B/RPR1B	Regulation of nuclear pre-mRNA domain-containing protein 1B	Transcription, Cell cycle
	O00422	SAP18	Histone deacetylase complex subunit SAP18	RNA processing and splicing, Transcription
	P11387	TOP1	DNA topoisomerase 1	Chromatin remodeling, DNA replication

	Q99757	TXN2/THIOM	Thioredoxin, mitochondrial	Cellular response, Electron transport
**s-cal14.2b**				
	P02656	APOC3	Apolipoprotein C-III	Lipid metabolism and degradation
	P07384	CAPN1/CANP1	Calpain-1 catalytic subunit	Proteolysis, Mammary gland involution, Signal trasduction, Cytoskeleton remodeling
	Q96EY1	DNAJA3/DNJA3	DnaJ homolog subfamily A member 3, mitochondrial	Apoptosis, Cellular response, Mitochondrial DNA replication
	Q6P587	FAHD1	Acylpyruvase FAHD1, mitochondrial	Cellular metabolism
	P40939	HADHA/ECHA	Trifunctional enzyme subunit alpha, mitochondrial	Lipid metabolism
	P68871	HBB	Hemoglobin subunit beta	Oxigen transport
	P33947	KDELR2/ERD22	ER lumen protein-retaining receptor 2	Protein transport
	Q9UHB6	LIMA1	LIM domain and actin-binding protein 1	Lipid metabolism, Steroid metabolism, Cell migration, Cytoskeleton modeling
	Q9UPQ0	LIMCH1/LIMC1	LIM and calponin homology domains-containing protein 1	Cytoskeleton organization, Cellular migration
	O14880	MGST3	Microsomal glutathione S-transferase 3	Lipid metabolism
	Q9Y3D9	MRPS23/RT23	28s ribosomal protein S23, mitochondrial	Undefined

	P53801	PTTG1IP/PTTG	Pituitary tumor-transforming gene 1 protein-interacting protein	Protein regulator
	P09417	QDPR/DHPR	Dihydropteridine reductase	Cellular response and metabolism
	O00194	RAB27B/RB27B	Ras-related protein Rab-27B	GTPasa activity
	Q9BWM7	SFXN3	Sideroflexin-3	Aminoacid transport
	P22695	UQCRC2/QCR2	Cytochrome b-c1 complex subunit 2, mitochondrial	Energy production, Electron transport, Proteolysis, Response to drugs
	Q7Z2W4	ZC3HAV1/ZCCHV	Zinc finger CCCH-type antiviral protein 1	Immune response
	P27701	CD82	CD82 antigen	Immune response
	Q16643	DBN1/DREB	Drebrin	Cellular differentiation, Cytoskeleton organization, Cell proliferation
	Q9C0B1	FTO	Alpha-ketoglutarate-dependent dioxygenase FTO	DNA repair, RNA repair, DNA demethylation
	O00214	LGALS8/LEG8	Galectin-8	Autophagy, Cellular migration
	Q92542	NCSTN/NICA	Nicastrin	Cell homeostasis
	P16435	POR/NCPR	NADPH-citocromo P450 reductasa	Electron transport
	Q14257	RCN2	Reticulocalbin-2	Unknown
	P46779	RPL28/RL28	60S ribosomal protein L28	Translation

	Q9NQG5	RPRD1B/RPR1B	Regulation of nuclear pre-mRNA domain-containing protein 1B	Transcription, Cell cycle
	Q9UGP8	SEC63	Translocation protein SEC63 homolog	Protein transport
	P09661	SNRPA1/RU2A	U2 small nuclear ribonucleoprotein A´	mRNA processing and splicing
	O60493	SNX3	Sortin nexin-3	Protein transport
	P23193	TCEA1	Transcription elongation factor A protein 1	RNA transcription
Shaded cells indicate subexpressed proteins; all functions indicated in biological process were obtained from uniprotkB protein database.				

REF 28. UniProt Consortium. UniProt: the universal protein knowledgebase in 2021. Nucleic Acids Res. 2021 Jan 8;49(D1): D480-D489. doi: 10.1093/nar/gkaa1100. PMID: 33237286; PMCID: PMC7778908.
