# Supplementary material for: Preclinical Efficacy and Proteomic Prediction of Molecular Targets for s-cal14.1b and s-cal14.2b Conotoxins with Antitumor Capacity in Xenografts of Malignant Pleural Mesothelioma
Source: Mar Drugs. 2025 Jan 10;23(1):32. doi: 10.3390/md23010032 (PMC11767107; doi:10.3390/md23010032)
Supplement: Supplementary file 1 [file marinedrugs-23-00032-s001.zip › marinedrugs-3281450-supplementary/Table S5.pdf]

Table S5. Proteins modified by conotoxins during growth time of H2452 spheroids.

Conotoxin	ID protein	Abreviattion	Name	Biological process
**s-cal14.1b**				
	P08133	ANXA6|	Annexin A6	Apoptosis, Mitochndrial homeostasis
	Q9H5V8	CDCP1	CUB domain-containing protein 1	Unknown
	Q15417	CNN3	Calponin-3	Cytoskeleton organization
	Q16643	DBN1/DREB	Debrin	Cellular differentiation, Cytoskeleton organization, Cell proliferation
	Q9NVH1	DNAJC11/DJC11	DnaJ homolog subfamily C member 11	inner mitochondrial membrane organization
	Q96CS3	FAF2	FAS-associated factor 2	response to unfoldeed protein
	Q16775	HAGH/GLO2	Hydroxyacylglutathione hydrolase, mitochondrial	energy production
	Q7Z6Z7-2	HUWE1	E3 ubiquitin-protein ligase HUWE1	cell differentiation, DNA damage and repair, protein degratation
	P51553	IDH3G	Isocitrate dehydrogenase [NAD] subunit gamma, mitochondrial	Energy production
	P12268	IMPDH2/IMDH2	Inosine-5´-monophosphate dehydrogenase 2	Purine biosyntesis,
	Q6IAA8	LAMTOR1/LTOR1	Regulator complex protein LAMTOR1	Cell cycle, Cellular response

	O14880	MGST3	Microsomal glutathione S-trnasferase 3	Lipid metabolism
	P28331	NDUFS1/NDUS1	NADH-ubiquinone oxidoreductase 75 kDa subunit, mitochodrial	Eelectron transport, Energy production
	O94903	PROSC/PLPHP	Pyridoxal phosphate homeostasis protein	Undefined
	O15126	SCAMP1/SCAM1	Secretory carrier-assiated membrane protein 1	Protein transport
	P84022	SMAD3	Mothers agains decapentaplegic homolog 3	DNA transcription, Cell migration
	Q9NYL9	TMOD3	Tropomodulin-3	Cytoskeleton organization, Cell cycle
	P46379	BAG6	Large proline-rich protein BAG6	Apoptosis, Cellular differentiation, Immune response, Protein transport
	Q96A33	CCDC47/CCD47	Coiled-coil domain-containing protein 47	Cellular organization
	P01892	HLA-A	HLA class I histocompatibility antigen, A alpha chain	Immune response
	Q12906-7	ILF3	Interleukin enhancer-binding factor 3	Transcription, Antiviral defense
	Q86UP2	KTN1	Kinectin	Cellular metabolism, Protein transport

	Q96RS6	NUDCD1	NudC domain-containing protein 1	Immune response
	Q9BTU6	PI4K2A/P4K2A	Phosphatidylinositol 4-kinase type 2-alpha	Protein modification and transport
	P17980	PSMC3/PRS6A	26S proteasome regulatory subunit 6A	Protein modification and transport
	P47897	QARS1/SYQ	Glutamine-tRNA ligase	Protein biosynthesis
	P27694	RPA1/RFA1	Replication protein A 70 kDa DNA-binding subunit	DNA damage, recombination, repair and replication
	P39019	RPS19/RS19	40S ribosomal protein S19	RNA translation
	Q92504	SLC39A7/S39A7	Zinc transporter SLC39A7	Ion transport
	P09234	SNRPC/RU1C	U1 small nuclear ribonucleoprotein C	RNA splicing
	Q15388	TOMM20/TOM20	Mitochondrial import receptor subunit TOM20 homolog	Protein transport
**cal14.2b**				
	P11310-2	ACADM	Medium-chain specific acyl-CoA dehydrogenase, mitochondrial	Lipid metabolism

	P24752	ACAT1/THIL	Acetyl-CoA acetyltransferase, mitochondrial	Lipid metabolism
	P27144	AK4/KAD4	Adenylate kinase 4, mitochondrial	Energy production, Cellular response
	P36542	ATP5C1/ATPG	ATP synthase subunit gamma, mitochondrial	Energy production, Ion transport
	Q9HB07	C12orf10/MYG1	MYG1 exonuclease	Cellular exploratory moving
	P07108	DBI/ACBP	Acyl-CoA-binding protein	RNA transcription
	P33316	DUT	Deoxyuridine 5'-triphosphate nucleotidohydrolase, mitochondrial	Nucleotide metabolism
	Q52LJ0-2	FAM98B/FA98B	Protein FAM98B	Protein modification
	Q16774	GUK1/KGUA	Guanylate kinase	Cellular metabolism
	P55084	HADHB/ECHB	Trifunctional enzyme subunit beta, mitochondrial	Lipid metabolism
	Q6IAA8	LAMTOR1/LTOR1	Regulator complex protein LAMTOR1	Cell cycle, Cellular response
	Q3KQU3-4	MAP7D1/MA7D1	MAP7 domain-containing protein 1	Cytoskeleton organization
	P28331	NDUFS1/NDUS1	NADH-ubiquinone oxidoreductase 75 kDa subunit, mitochodrial	Electron transport, Energy production

	Q9H0P0	NT5C3A/5NT3A	Cytosolic 5'-nucleotidase 3A	Nucleotide metabolism
	O00151	PDLIM1/PDLI1	PDZ and LIM domain protein 1 (Elfin)	Response to hypoxia, RNA transcription, Cytoskeleton organization
	P30405	PPIF	Peptidyl-prolyl cis-trans isomerase F, mitochondrial (cyclophilin D)	Apoptosis, Necrosis
	P08134	RHOC	Rho-related GTP-binding protein RhoC	Signal transduction, Cell adhesion
	P62273	RPS29/RS29	40S ribosomal protein S29	mRNA processing and splicing
	P84022	SMAD3	Mothers agains decapentaplegic homolog 3	DNA transcription, Cell migration
	Q8IYB3	SRRM1	Serine/arginine repetitive matrix protein 1	mRNA processing and splicing
	Q8NC54	KCT2	Keratinocyte-associated transmembrane protein 2	Undefined
	Q92974	ARHGEF2/ARHG2	Rho guanine nucleotide exchange factor 2	Cell cycle and division, Cellular response and differentiation, Immune response, Protein transport
	Q13620	CUL4B	Cullin-4B	Cell cycle, DNA repair and damage, Protein modification
	P31689	DNAJA1	DnaJ homolog subfamily A member 1	Cellular response
	Q7Z6Z7-2	HUWE1	E3 ubiquitin-protein ligase HUWE1	Cell differentiation, DNA damage and repair, Protein degradation
	O95373	IPO7	Importin-7	Protein transport, Immune response

	P08473	MME/NEP	Neprilysin	Cellular response, Kidney and lung development
	P22059	OSBP/OSBP1	Oxysterol-binding protein 1	Lipid transport
	Q969U7	PSMG2	Proteasome assembly chaperone 2	Apoptosis
	P51148	RAB5C	Ras-related protein Rab-5C	Protein transport
	P84098	RPL19/RL19	60S ribosomal protein L19	RNA transcription
	P60866	RPS20/RS20	40S ribosomal protein S20	RNA transcription
	Q15637	SF1/SF01	Splicing factor 1	RNA transcription, splicing and processing
	P43307	SSR1/SSRA	Translocon-associated protein subunit alpha	Protein translocation
	P0DMN0	SULT1A4/ST1A4	Sulfotransferase 1A4	Lipid metabolism
	Q01081	U2AFBP/U2AF1	Splicing factor U2AF 35 kDa subunit	RNA transcription, splicing and processing
	P20742	PZP	Pregnancy zone protein	Undefined

Shaded cells indicate subexpressed proteins; all functions indicated in biological process were obtained from uniprotkB protein data base.

REF 28. UniProt Consortium. UniProt: the universal protein knowledgebase in 2021. Nucleic Acids Res. 2021 Jan 8;49(D1): D480-D489. doi: 10.1093/nar/gkaa1100. PMID: 33237286; PMCID: PMC7778908.
