# Supplementary figures and images for: Preclinical Efficacy and Proteomic Prediction of Molecular Targets for s-cal14.1b and s-cal14.2b Conotoxins with Antitumor Capacity in Xenografts of Malignant Pleural Mesothelioma
Source: Mar Drugs. 2025 Jan 10;23(1):32. doi: 10.3390/md23010032 (PMC11767107; doi:10.3390/md23010032)

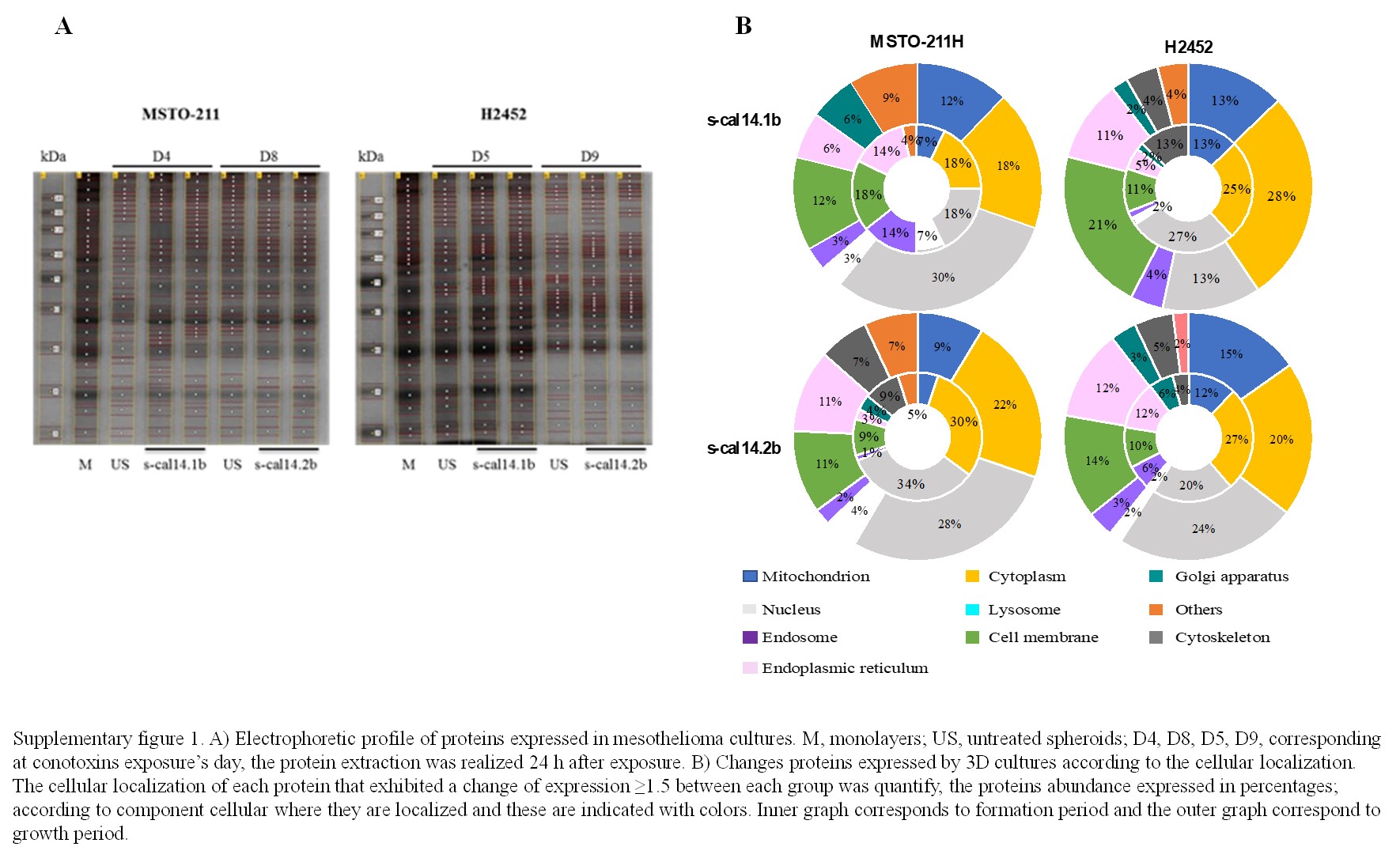

Supplement: Supplementary file 1 [file marinedrugs-23-00032-s001.zip › marinedrugs-3281450-supplementary/Figure 1.jpg]

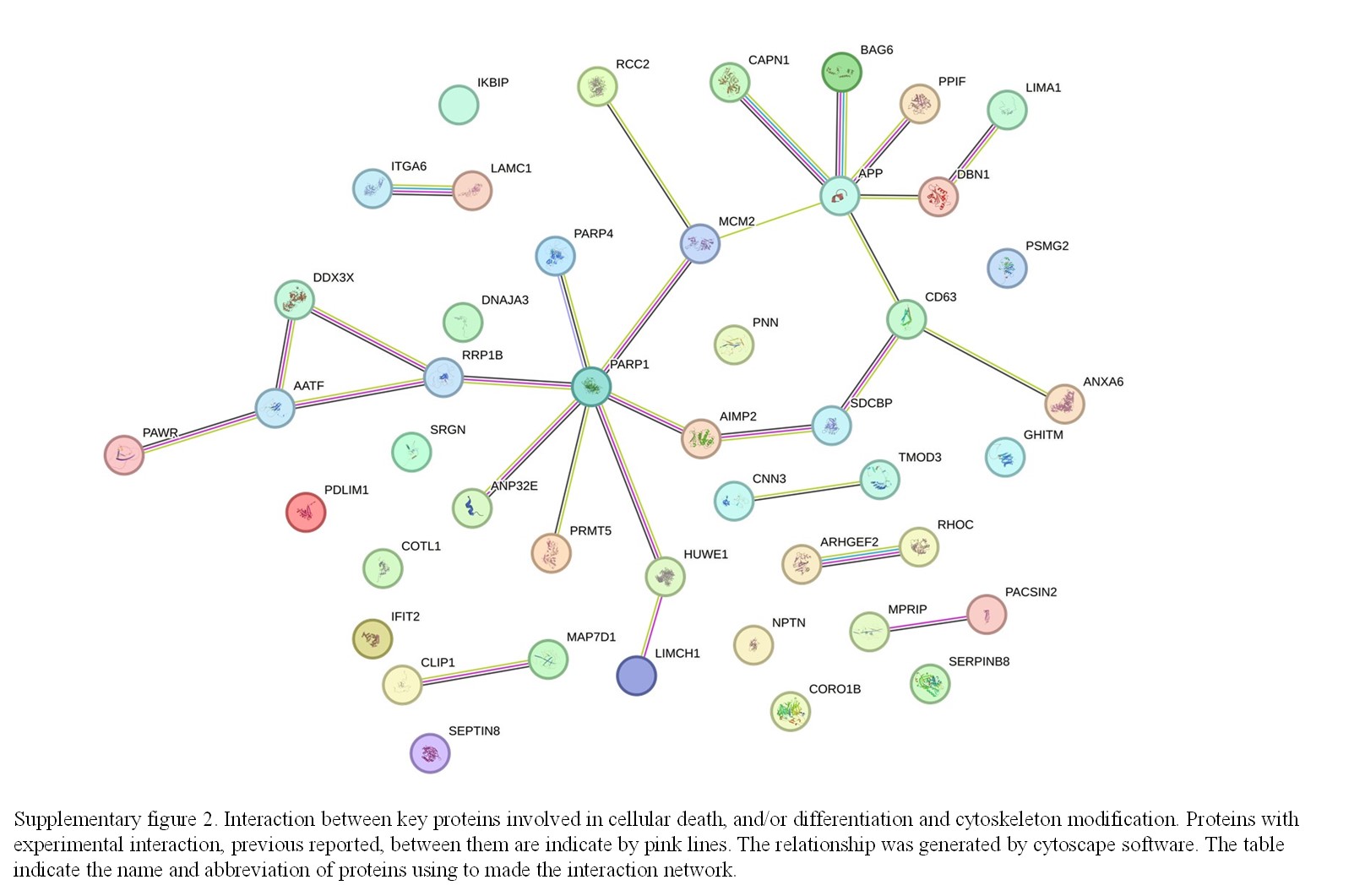

Supplement: Supplementary file 1 [file marinedrugs-23-00032-s001.zip › marinedrugs-3281450-supplementary/Figure 2.jpg]
